# Causal relationships of familial hypercholesterolemia with the risk of multiple vitamin deficiencies: a Mendelian randomization study

**DOI:** 10.3389/fendo.2024.1401260

**Published:** 2024-10-22

**Authors:** Cheng Zhang, Gang Wei, Huan Zhou, Lin Liu

**Affiliations:** ^1^ National Drug Clinical Trial Center, The First Affiliated Hospital of Bengbu Medical University, Bengbu, Anhui, China; ^2^ Beijing Key Laboratory of Diabetes Research and Care, Department of Endocrinology, Beijing Diabetes Institute, Beijing Tongren Hospital, Capital Medical University, Beijing, China

**Keywords:** familial hypercholesterolemia, genetic association, human, thiamine deficiency, vitamin D deficiency

## Abstract

**Background:**

The causal relationship between familial hypercholesterolemia (FH) and various vitamin deficiencies has not yet been elucidated. Therefore, this study investigated the cause-and-effect relationship between FH and the risk of multiple vitamin deficiencies in humans.

**Methods:**

Mendelian randomization (MR) analysis was performed by extracting six datasets for FH, FH with ischemic heart disease (IHD), and vitamin deficiency (vitamin A, thiamine, other B-group vitamins, and vitamin D) from the FinnGen study, covering a total of 329,115; 316,290; 354,932; 354,949; 355,411 and 355,238 individuals, respectively.

**Results:**

FH was suggestively associated with higher odds of thiamine deficiency [inverse variance weighted odds ratio (OR_IVW_) 95% confidence interval (CI): 1.62 (1.03, 2.55), *P* = 0.036] and vitamin D deficiencies [OR_IVW_ CI: 1.35 (1.04, 1.75), *P* = 0.024], low-density lipoprotein receptor (*LDLR*) rs112898275 variant, rs11591147 and rs499883 in proprotein convertase subtilisin/kexin 9 (*PCSK9*), rs9644862 in cyclin-dependent kinase inhibitor 2 B antisense RNA1 (*CDKN2B-AS1*), and rs142834163 in dedicator of cytokinesis 6 (*DOCK6*) and rs115478735 in ABO blood group (*ABO*) strongly influenced the risk of thiamine deficiency, while the rs7412 variant in apolipoprotein E (*APOE*) mostly influenced the risk of vitamin D deficiency. FH with IHD was suggestively associated with higher odds of vitamin D deficiency (OR_IVW,_ weighted median [WM][95%CI]: 1.31 [1.05, 1.64]; 1.47 [1.10, 1.97]) (*P* = 0.018; 0.010) without any single significant SNPs observed.

**Conclusion:**

FH was positively associated with increased risks of thiamine and vitamin D deficiencies, revealing a prospective and unfortunate complication of FH.

## Introduction

Vitamins are a cluster of organic substances that sustain the growth, reproduction, and homeostasis of the human body (1). They exist as fat-soluble (vitamins A, D, E, and K) and water-soluble (vitamins B and C) vitamin forms (2). Except for vitamin D, which can be synthesized by humans, the other vitamins are obtained from food intake and gut microbiota (2, 3). Vitamin deficiency is common worldwide at all ages (4). Minor vitamin deficiencies are insidious and often overlooked in the clinic, but can have severe negative effects (4). The most common examples of these effects include xerophthalmia, anemia, a high incidence rate of infectious diseases (vitamin A), beriberi (thiamine or vitamin B1), rickets, osteomalacia, and a possible association with increased infectious diseases (vitamin D) (4). Globally, vitamin A deficiency occurs in nearly 30% of children <5 years of age. The morbidity of thiamine deficiency is 20%–90%, and the regional epidemic rate of vitamin D deficiency is 5.5–85.1% (5–7). Vitamin deficiency has become an important public health issue due to its high prevalence and undesirable endpoints (8).

Familial hypercholesterolemia (FH) is a congenital mal-transportation of lipids bearing homozygous or heterozygous family mutated genes, which is marked by a superelevation of plasma low-density lipoprotein cholesterol (LDL-C) levels (9). This overload can genetically accelerate vitamin D deficiency (10). Additionally, it has been reported from observational investigation that vitamins D, B6, and B12 deficiencies occurred in hyperlipidemic patients, with vitamins B6 significantly negatively associated with total cholesterol and non-HDL levels (11). Hence, patients with FH are likely complicated with multiple vitamin deficiencies, spanning the entire course of disease. However, the known Mendelian effect of high LDL-C levels on vitamin D deficiency has significant pleiotropy (10); meanwhile, the observational investigation has the sample size of only 60 (including 40 hyperlipidemic patients and 20 healthy controls), and the subjects of only males from Jordanian (11). Therefore, the Mendelian model of FH can be applied to assess the future risk of multiple vitamin deficiencies to ensure its prevention in the background of FH.

Mendelian randomization (MR) design assessed the possible causal relationship between FH and the risk of multiple vitamin deficiencies, including vitamin A, thiamine, other B-group vitamins, and vitamin D.

## Materials and methods

**Figure 1:**
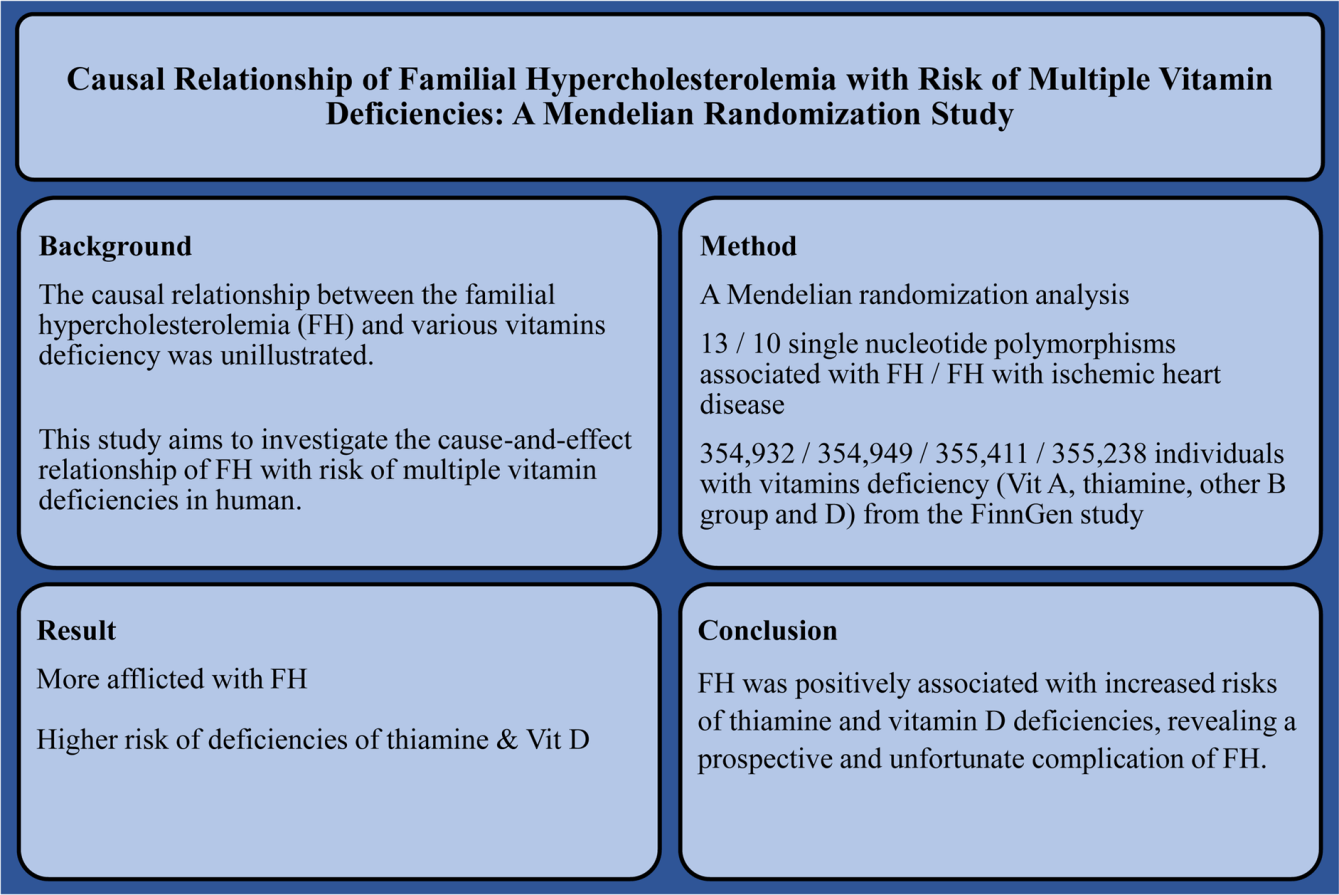
Graphical abstract of this study.

**Figure 2:**
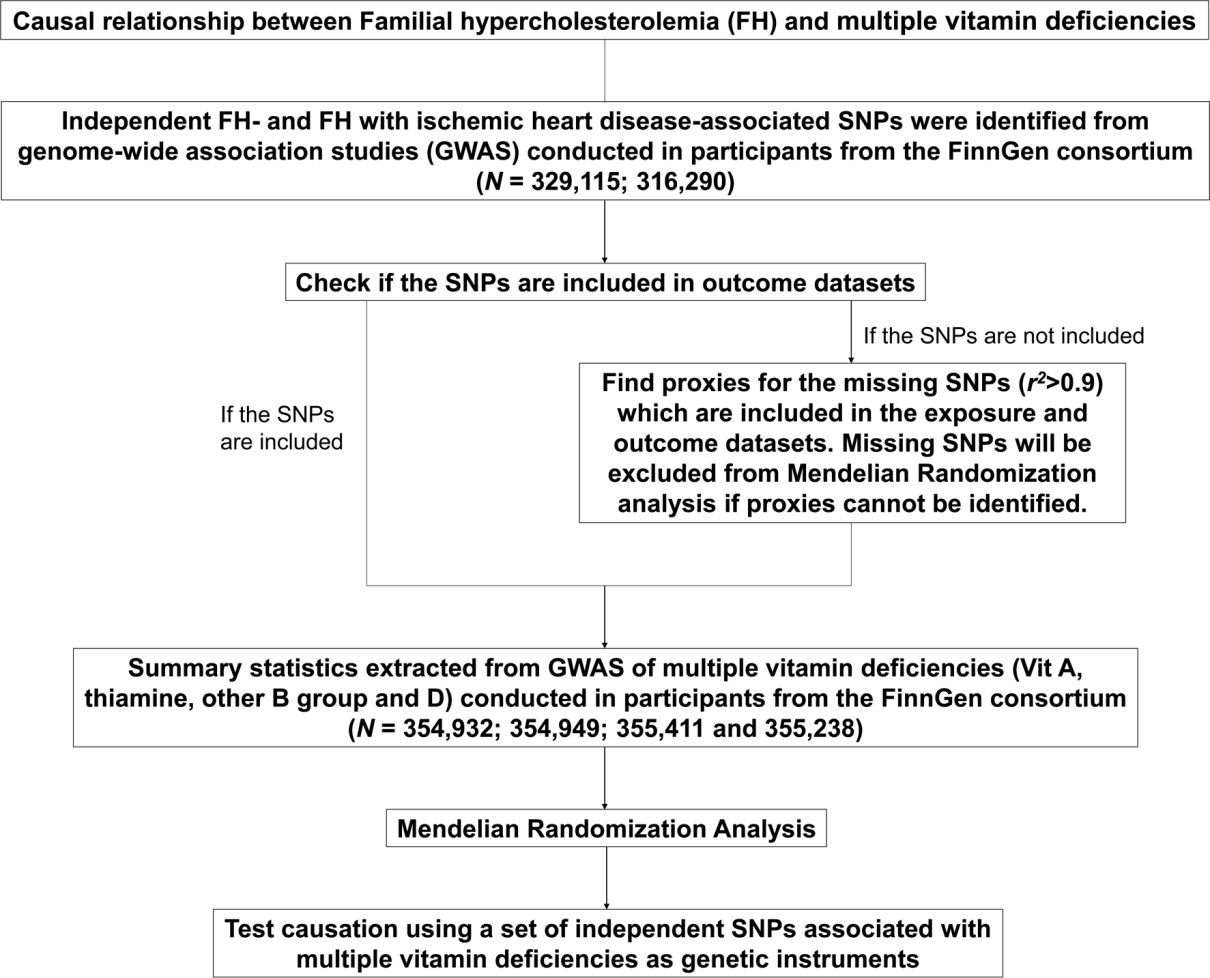
Flowchart of the Mendelian randomization analyses of familial hypercholesterolemia and the risk of multiple vitamin deficiencies. FH, familial hypercholesterolemia.

**Figures 1**, **2** show the graphical abstract and an overview of the study design, respectively. An MR design based on public summary-level data derived from genome-wide association studies (GWASs) was adopted to evaluate the possible causal relationship between FH and ischemic heart disease (IHD) with the risk of multiple vitamin deficiencies (vitamin A, thiamine, other B-group vitamins, and vitamin D).

### Instrumental variable selection

Genetic variants of FH (ICD10: E7800) and FH with IHD were obtained from a recently published GWAS of European ancestry (FinnGen) (*N* = 329,115; 316,290) (**Supplementary Table S2**). Summary datasets are available on the following websites (https://storage.googleapis.com/finngen-public-data-r9/summary_stats/finngen_R9_E4_FH.gz and https://storage.googleapis.com/finngenpublic-data-r9/summary_stats/finngen_R9_E4_FH_IHD.gz). In the MR analysis, IVs were chosen on the basis of specific criteria. Single nucleotide polymorphisms (SNPs) associated with FH and FH with IHD were selected at a genome-wide significance level of *P* < 5×10^−8^. When the *r^2^* of SNPs was < 0.001 within a 10000 kb window, the SNP with a more significant *P*-value was retained from the analysis. SNPs not available in the vitamin deficiency datasets were either replaced with proxy SNPs in high linkage disequilibrium (LD, *r^2^* > 0.9) or discarded. Finally, palindromic SNPs were discarded based on their allele frequencies.

The use of strong instruments is key to improving the accuracy and efficiency of estimating causal effects in the MR model. However, if the genetic variation is only weakly linked to the exposure variable, it may introduce bias in the estimates of the MR model, which is commonly referred to as weak instrument bias (12). We further assessed the strength of genetic variants by computing the F-statistic (F = *β* ^2^/SE^2^) for each SNP, ensuring that the F-statistic exceeded 10 (12, 13). In this study, the minimum F-statistic observed was 30.85, suggesting strong instruments and consequently a low likelihood of bias from weak instruments. **Supplementary Table S1** provides an overview of these data.

### Outcome data source

Genetic variants of multiple vitamin deficiencies (vitamin A (ICD10: E50), thiamine (ICD10: E51), other B-group vitamins (ICD10: E53), and vitamin D (ICD10: E55)) were obtained from FinnGen (*N* = 354,932; 354,949; 355,411; and 355,238, respectively). The summary dataset is available on the website (https://storage.googleapis.com/finngen-public-data-r9/summary_stats/finngen_R9_E4_VIT_A_DEF.gz; https://storage.googleapis.com/finngen-public-data-r9/summary_stats/finngen_R9_E4_THIA_DEF.gz; https://storage.googleapis.com/finngen-public-data-r9/summary_stats/finngen_R9_E4_VIT_B_DEF.gz; https://storage.googleapis.com/finngen-public-data-r9/summary_stats/finngen_R9_E4_VIT_D_DEF.gz). **Supplementary Table S2** provides detailed information.

### Statistical analyses

The MR method was used to explore the causal associations between genetically predicted differences per standard deviation (SD) increases in FH and the risk of vitamin deficiency by reporting odds ratios (ORs). The conventional inverse-variance weighted (IVW) and weighted median (WM) methods were used in the primary analysis (14). Cochran’s Q test using the IVW model was applied to quantify heterogeneity (15). The MR-Egger regression model was used to determine unknown pleiotropic effects. A non-zero intercept from MR-Egger indicates that the IVW estimate may be invalid due to horizontal pleiotropy (16). Sensitivity analysis based on the MR-Egger regression model and leave-one-out sensitivity analysis were performed (16). Furthermore, the PhenoScanner database was searched to assess the association of the selected SNPs with possible pleiotropy at a genome-wide significance level of *P <*5×10^−8^ *(* 17, 18). To strengthen the reliability of the results, this study assessed the statistical power of significant associations. This calculation determined the probability of detecting a true effect in this MR study, considering the specified sample size and effect size (19, 20).

All statistical analyses were conducted using R software (R 4.0.5, The R Foundation for Statistical Computing) and the “TwoSampleMR” package (21). The Bonferroni correction is a conservative method for probability thresholding to control the occurrence of false positives. To account for multiple testing, the Bonferroni correction threshold of *P*-value < 6.25 × 10^-3^ (0.05/8 [2 exposures and 4 outcomes]) was prespecified. Significance was determined at a *P*-value < 6.25 × 10^-3^, while the *P*-values between 6.25 × 10^-3^ and 0.05 was considered suggestive (22, 23). For analyses of heterogeneity and pleiotropy, *P*-value < 0.05 indicated significant (22).

## Results

### Genetic variants associated with familial hypercholesterolemia

The genetic tool extraction identified 13 SNPs related to FH, and 10 SNPs related to FH with IHD (all *P* < 5×10^−8^, *r^2^* < 0.001, 10000 kb) (**Supplementary Table S1**).

### Cause-and-effect relationship of familial hypercholesterolemia with multiple vitamin deficiencies

The primary results of IVW and WM estimated the causal relationship between FH/FH with IHD and multiple vitamin deficiencies. The outcomes of IVW model suggested positive genetic causal associations between FH and deficiencies of thiamine and vitamin D, respectively (OR [95%CI]: 1.62 [1.03, 2.55]; 1.35 [1.04, 1.75]) (*P* = 0.036; 0.024). Similarly, the outcomes of IVW and WM models suggested positive genetic causal association between FH with IHD and vitamin D deficiency (OR [95%CI]: 1.31 [1.05, 1.64]; 1.47 [1.10, 1.97]) (*P* = 0.018; 0.010) (**Table 1**).

**Table 1:** Inverse variance-weighted and weighted median analyses of familial hypercholesterolemia and risk of multiple vitamin deficiencies.

Exposure	Outcome	Inverse variance-weighted method			Weighted median method		
		SNPs, n	OR (95%CI)	*P*-value	SNPs, n	OR (95%CI)	*P*-value
Familial hypercholesterolemia	Vitamin A deficiency	13	0.68 (0.42, 1.12)	0.13	13	0.93 (0.48, 1.77)	0.82
	Vitamin thiamine deficiency	13	1.62 (1.03, 2.55)	0.036	13	1.61 (0.85, 3.06)	0.15
	Deficiency of other B group vitamins	13	0.91 (0.73, 1.13)	0.38	13	0.90 (0.68, 1.21)	0.49
	Vitamin D deficiency	13	1.35 (1.04, 1.75)	0.024	13	1.21 (0.86, 1.72)	0.28
Familial hypercholesterolemia, with ischemic heart disease	Vitamin A deficiency	10	0.99 (0.65, 1.51)	0.96	10	1.01 (0.58, 1.77)	0.97
	Vitamin Thiamine deficiency	10	1.40 (0.94, 2.07)	0.10	10	1.56 (0.92, 2.65)	0.10
	Deficiency of other B group vitamins	10	0.96 (0.79, 1.16)	0.67	10	0.95 (0.73, 1.22)	0.67
	Vitamin D deficiency	10	1.31 (1.05, 1.64)	0.018	10	1.47 (1.10, 1.97)	0.010

In addition, no causal relationships showed between FH and the risks of deficiencies in vitamin A and other B-groups vitamins, and between FH with IHD and the risks of deficiencies in vitamin A, thiamine, and other B-group vitamins (*P* > 6.25 × 10^-3^) (**Table 1**).

### Sensitivity analysis of familial hypercholesterolemia with multiple vitamin deficiencies

Sensitivity analysis was conducted on the suggestive exposure-outcome associations obtained to verify their reliability. No significant heterogeneity (*P* = 0.52) and horizontal pleiotropy (*P* = 0.40) were observed in the correlation analysis between FH and thiamine deficiency. No significant heterogeneity (*P* = 0.88) and horizontal pleiotropy (*P* = 0.28) were observed in the correlation analysis between FH and vitamin D deficiency. No significant heterogeneity (*P* = 0.55) or horizontal pleiotropy (*P* = 0.62) was observed in the correlation analysis between FH with IHD and vitamin D deficiencies (**Table 2**).

**Table 2:** Cochran’s Q test, MR–Egger intercept and MR-Egger regression of familial hypercholesterolemia and risk of multiple vitamin deficiencies.

Exposure	Outcome	Cochran’s Q test^†^	MR-Egger intercept^‡^			MR-Egger regression			
		*P*-value	*β*	SE	*P*-value	SNPs, n	*β*	SE	*P*-value
Familial hypercholesterolemia	Vitamin A deficiency	0.60	0.06	0.11	0.61	13	-0.63	0.53	0.26
	Vitamin thiamine deficiency	0.52	0.09	0.10	0.40	13	0.11	0.48	0.83
	Deficiency of other B group vitamins	0.58	-0.03	0.05	0.53	13	0.03	0.23	0.89
	Vitamin D deficiency	0.88	0.07	0.06	0.28	13	0.02	0.28	0.94
Familial hypercholesterolemia, with ischemic heart disease	Vitamin A deficiency	0.52	0.10	0.15	0.54	10	-0.37	0.60	0.56
	Vitamin thiamine deficiency	0.64	-0.003	0.14	0.98	10	0.34	0.55	0.55
	Deficiency of other B group vitamins	0.84	-0.02	0.07	0.83	10	0.01	0.26	0.96
	Vitamin D deficiency	0.55	0.04	0.08	0.62	10	0.12	0.31	0.71

^†^The Cochran’s Q test is a statistical test for heterogeneity.

^‡^The intercept term from the MR–Egger regression method is a statistical test of horizontal pleiotropy.

**Figure 3:**
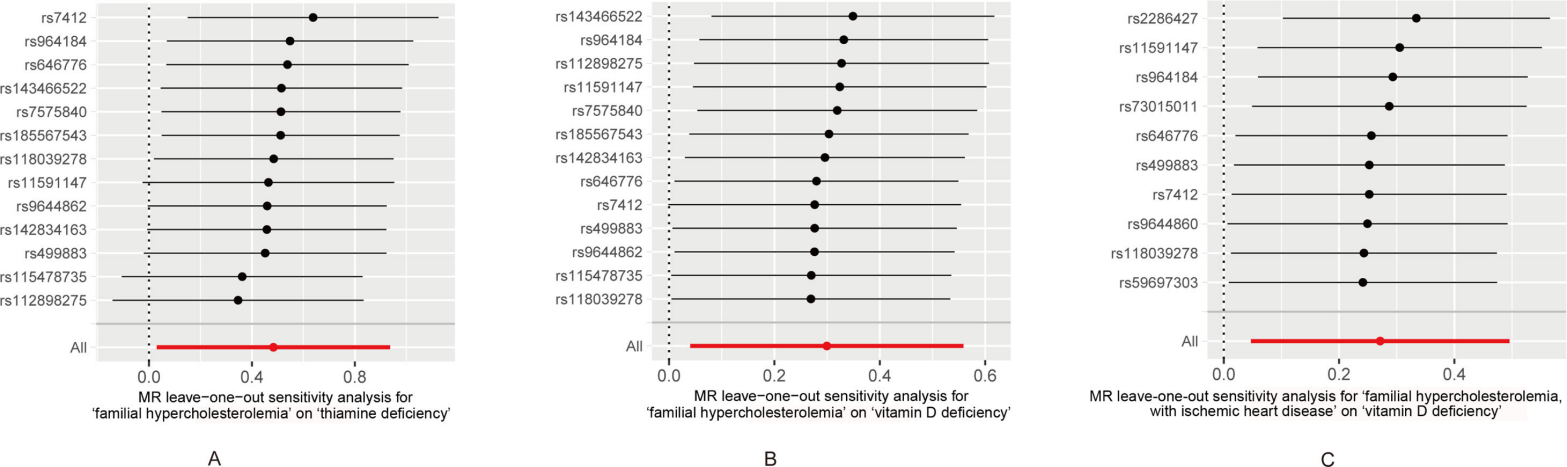
**(A)** Leave-one-out sensitivity and Mendelian randomization analyses based on the inverse-variance weighted (IVW) model for determining the effects of familial hypercholesterolemia on the risk of thiamine deficiency. **(B)** Leave-one-out sensitivity and Mendelian randomization analyses based on the inverse-variance weighted (IVW) model for determining the effects of familial hypercholesterolemia on the risk of vitamin D deficiency. **(C)** Leave-one-out sensitivity and Mendelian randomization analyses based on the inverse-variance weighted (IVW) model for determining the effects of familial hypercholesterolemia with ischemic heart disease on the risk of vitamin D deficiency.

The leave-one-out analysis was used to analyze single instrumental variable influencing the causal effects of FH/FH with IHD on multiple vitamin deficiencies. Leaving rs112898275, rs115478735, rs11591147, rs142834163, rs499883, and rs9644862 out respectively abrogated the correlation of FH with thiamine deficiency (*β* = -0.32103, 0.141933, -0.50812, 0.508043, 0.144529, 0.115318) (**Figure 3A**; **Supplementary Table S1**). Leaving rs7412 out, the correlation of FH with vit D deficiency did not remain (*β* = -0.42272) (**Figure 3B**; **Supplementary Table S1**). However, the correlation between FH with IHD and vitamin D deficiency remained even after removing any single SNPs (**Figure 3C**).

Moreover, the statistical power of FH and thiamine/vitamin D deficiencies were 50%/49%, and FH with IHD and vitamin D deficiency was 49%.

## Discussion

**Figure 4:**
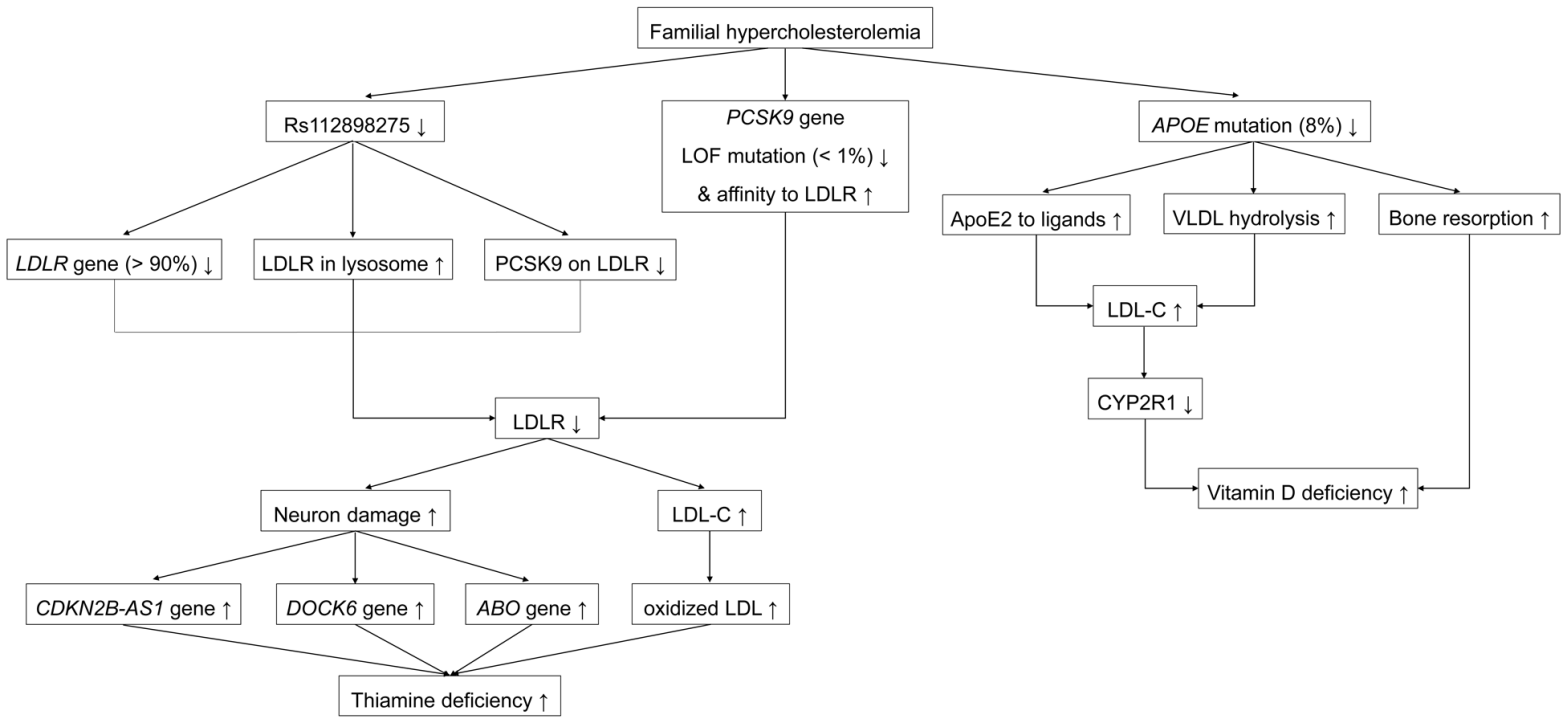
Representative signaling pathways involved in the associations between familial hypercholesterolemia and multiple vitamin deficiencies. *ApoE*, apolipoprotein E; *PCSK9*, proprotein convertase subtilisin/kexin 9; *CDKN2B-AS1*, cyclin-dependent kinase inhibitor 2B antisense RNA1; LOF, loss of function; GOF, gain of function; *LDLR*, low-density lipoprotein receptor.

This study is the first to evaluate the causal effect of FH on the development of multiple vitamin deficiencies. The results of the MR analysis suggested positive associations between FH and thiamine and vitamin D deficiencies. Unlike FH, FH with IHD was only positively associated with higher odds of vitamin D deficiency. Significant associations were further tested using a leave-one-out sensitivity analysis of the mechanism. The typical signals are depicted in **Figure 4**.

### Cause-and-effect relationship of familial hypercholesterolemia with thiamine deficiency

Rs112898275 is a SNP in the low-density lipoprotein receptor (*LDLR*) gene shown in the Phenoscanner database. *LDLR* is 45 kb long, is located on the short arm of chromosome 19p13.2, and includes 18 exons and 17 introns (24–26). *LDLR* gives rise to the LDLR protein comprising 860 amino acids harboring a 21-amino acid signal peptide (26). The LDLR protein is processed in the endoplasmic reticulum (ER), where the signal peptide is cleaved; the cleaved protein is then transformed into a mature 160-kDa (839 amino acids) glycoprotein by glycosylation (25). Mature LDLR is a transmembrane receptor located on most cellular surfaces and includes LDL receptor type A (LA) repeats, an epidermal growth factor precursor (EGFP), a transmembrane section, and a cytoplasmic tail with at least one NPxY theme (25, 27). The LA repeats bind to LDL-C on the cell membrane, and EGFP facilitates the combination of LDLR with proprotein convertase subtilisin/kexin 9 (PCSK9) on the hepatocellular surface (primary) and in the trans-Golgi network (TGN) (secondary), and liberation of bound LDL-C in the endosome, whereby LDLR/PCSK9 and released LDL-C are targeted for lysosomal degradation and non-adherent LDLR returns to the cell surface for reutilization (27, 28). *LDLR* mutagenesis accounts for > 90% of mutations in FH (29). The inverse *β*-coefficients of rs112898275 lead us to hypothesize that increased *LDLR* rs112898275 in patients with FH diminishes LDLR reutilization or adhesion to PCSK9.

Rs11591147 and rs499883 are SNPs in the proprotein convertase subtilisin/kexin 9 (*PCSK9*) gene (30). *PCSK9* is located on the small arm of chromosome 1p32 and has a length of 25 kb, 12 exons, and 11 introns (28). The main organ producing PCSK9 is the liver, followed by the kidneys and intestines (28). *PCSK9* initially synthetizes the inactive 692-amino acid PCSK9 protein with a signal peptide (amino acids 1–30) (31). The PCSK9 protein is processed in the ER, where the signal peptide is removed and cleavage occurs autocatalytically at the VFAQ152-SIP site (28, 31). The cleaved mature protein is then delivered to the Golgi apparatus in eukaryotic cells (28, 32). Later, when dispersed in plasma, the catalytic domain (amino acids 153–421) of the mature PCSK9 protein links with EGFP (amino acids 314–355) of the LDLR on the cellular surface of liver cells (primary) and in the TGN (secondary) by protein-protein communication (28, 33). LDLR/PCSK9 travels into lysosomes from endosomes for degradation, resulting in inadequate LDLR recycling to the cytomembrane to eliminate LDL-C (25). The two contrary false expressions of *PCSK9* include loss-of-function (LOF), which decreases cholesterol, and gain-of-function (GOF), which increases cholesterol levels (34). Among FH mutations, < 1% are GOF mutations in *PCSK9*; the remaining >99% are non-GOF (29). The rs11591147 (p.Arg46Leu) is located in exon 1 of *PCSK9*; this LOF-mutation site impairs lysosomal degradation by reducing by 15% the connectivity of PCSK9 to LDLR, thereby combating high LDL-C (25, 28, 32, 35, 36). Meanwhile, rs499883 is an intronic SNP in *PCSK9* that positively regulates *PCSK9* expression via an unknown mechanism (30). Hence, decreased rs11591147 (p.Arg46Leu) and increased rs499883 in patients with FH leads to increased PCSK9 levels, which strengthens the binding of PCSK9 to LDLR for lysosomal degradation, resulting in insufficient LDLR for recycling.

Rs9644862 is a SNP in the cyclin-dependent kinase inhibitor 2 B antisense RNA1 (*CDKN2B-AS1*) gene (known as antisense non-coding RNA in the INK4 locus (*ANRIL*), *p15AS*, *PCAT12*, *CDKN2BAS*, *CDKN2B-AS*, *NCRNA00089*) gene (37–39). *CDKN2B-AS1* is located on the short arm of human chromosome 9p21.3 and has 21 exons (40, 41). CDKN2B-AS1, a product of *CDKN2B-AS1*, is a 3.8 kb non-coding RNA (lncRNA) (40, 41). CDKN2B-AS1 protects against neuronal apoptosis by increasing glial cell-derived neurotrophic factor (GDNF) levels and preventing neuronal apoptosis by absorbing micro-RNA (miR)-133 (42).

Rs142834163 is a SNP in the dedicator of cytokinesis 6 (*DOCK6*) gene, as shown in the Phenoscanner database. *DOCK6* (also known as AOS2 or ZIR1) is located on chromosome 19p13.2 and contains 51 exons (43). *DOCK6* encodes the 2047 amino acid DOCK6 protein (44). DOCK6 belongs to the DOCK family of guanine nucleotide exchange factors (GEFs), which promote the exchange of guanosine diphosphate (GDP) with guanosine triphosphate (GTP) to regulate Rho GTPase activity (45). DOCK6 has two DOCK homology region (DHR) domains with phospholipid-binding and membrane-targeting activity in DHR-1 and GEF in DHR-2 (46). During neurodevelopment, DOCK6 plays a role in neurite outgrowth, axon growth, and regeneration by exchanging GDP with GTP for ras-related C3 botulinum toxin substrate 1 (RAC1) and cell division cycle 42 (CDC42), which control lamellipodia and filopodia morphology, respectively (46–48).

Rs115478735, a SNP in *ABO*, is strongly associated with plasma proteins of the immunoglobulin superfamily, including leucine-rich repeat protein 2 (ISLR2) and protein-tyrosine sulfotransferase 2 (TPST2) (49). ISLR2 (also known as LINX) is a type I transmembrane protein and a subset of the leucine-rich repeat and immunoglobulin (LIG) family of proteins comprising five tandem leucine-rich repeats (LRRs), an immunoglobulin (IG) domain, a transmembrane domain, and a short cytoplasmic tail (50). ISLR2 uniquely determines axon extension, guidance, and branching by interacting with Trk receptor tyrosine kinases (RTKs) to regulate their activities (51). ISLR2 interacts with and enhances Rho-kinase activity to rebuild the cytoskeleton during neuronal development (50). TPST2 is a tyrosyl protein sulfotransferase (TPST), a membrane-bound enzyme that catalyzes the protein-tyrosine sulfation in the cellular TGN by transferring a sulfuryl group from 3’-phosphoadenosine 5’-phosphosulfate (PAPS) to tyrosine residues (52). Tyrosine sulfation plays an elusive role in neuronal growth and maintenance (52).

In the central nervous system (CNS), LDLR is predominantly located in neurons, astrocytes, and oligodendrocytes and blocks neuronal pyroptosis by hindering ROS-NLRP3 inflammasome activation (53). In animal models, the absence of *LDLR* increases ROS generation, vulnerability to amyloid-β (Aβ)-induced neurotoxicity, and caspase-1-dependent gasdermin D (GSDMD) cleavage, and leads to neuronal pyroptosis (53, 54). Besides, shortage of LDLR can also increases blood LDL levels for more LDL oxidation (25, 55). Therefore, patients with FH who lack LDLR naturally develop neuronal dysfunction and elevation of serum oxidized low-density lipoprotein (ox-LDL) levels.

Thiamine is a water-soluble vitamin; Thiamine is present in meat, beef, pork, legumes, whole grains, and nuts; however, milled rice and grains contain small amounts of thiamine because the processing involved in creating these food products removes thiamine. Additionally, food products such as tea, coffee, raw fish, and shellfish contain thiaminases that destroy thiamine (56). Intake thiamine, in cationic form of T^+^, is hydrolyzed by intestinal phosphatase into free form, following absorbed by small intestine (57, 58). Thiamine diphosphate (TDP), also named as Thiamine pyrophosphate (TPP), which is derived from T^+^ by thiamine pyrophosphokinase-1 (TPK1), is the main active form of Thiamine, playing as a co-enzyme for glucose, amino acid, and lipid metabolism (57, 58). Thiamine favors neuronal function by serving as a site-specific antioxidant, and promotes energy production by utilizing carbohydrates (59). At the same time, TPP prevents atherosclerosis by reducing macrophage uptake of ox-LDL via antagonizing macrophage P2Y6 receptor (60). In patients with FH, Thiamine may be required to not only repair secondary neuronal damage but also decrease macrophage uptake of excessive ox-LDL, both of which caused by a lack of LDLR. Thiamine deficiency begins when demand exceeds supply, and poor intake intensifies this damage. The effects of decreased rs112898275 and rs11591147, combined with increased rs115478735, rs142834163, rs499883, and rs9644862 on thiamine deficiency requires further investigation.

### Cause-and-effect relationship of familial hypercholesterolemia with vitamin D deficiency

Rs7412 is a SNP in the apolipoprotein E (*APOE*) gene (61). *APOE* resides on chromosome 19p13.32 and is 3,612 bp in length, including four exons and three introns (62). The liver is the dominant expresser of *ApoE*, which is less abundant in the brain, spleen, kidneys, gonads, adrenal glands, and macrophages (61). *APOE* originally generates the 317-amino acid apoE precursor, including an 18-amino acid signal peptide (62). Cleavage of this 18-amino acid signal peptide and glycosylation results in the conversion of the precursor into a mature 299-amino acid, 34,200-kDa protein (61). Apo appears in chylomicrons, very low-density lipoprotein (VLDL), intermediate-density lipoprotein (IDL), LDL, high-density lipoprotein (HDL), and lipoprotein (a) (Lp(a)), and connects to each corresponding receptor to clear them (61). ApoE has a 22-kDa receptor-binding amino-terminal domain (amino acids 1–191) near residues 136–150 and a 10-kDa lipid-binding carboxyl-terminal domain (amino acids 216–299) (63). The three subtypes—apoE2 (Cys112; Cys158), apoE3 (Cys112; Arg158), and apoE4 (Arg112; Arg158)—result from the APOE*ϵ2, APOE*ϵ3 and APOE*ϵ4 alleles, with rs7412 in exon 4 contributing to codon 158 (64–66). ApoE2 from rs7412 (p.Arg176Cys), deforms the salt bridge in the structure to reduce the positive potential in the receptor-binding region, which reduces the affinity of apoE2 to the LDLR, VLDL receptor (VLDLR), and LDLR-related protein (LRP) (61). In contrast, apoE2 rs7412 (p.Arg176Cys) prevents the hydrolysis of triglycerides in VLDL to form VLDL remnants (IDL) as LDL precursors by inhibiting lipoprotein lipase (LPL) on the capillary endothelium (61). This mutation accounts for nearly 8% of the population and results in an LDLR affinity of < 2% of normal, which represents as negativity for *β*-coefficient with LDL-C in FH, consistent with the findings of this study (67, 68). Thus, decreased rs7412 (p.Arg176Cys) in patients with FH increases LDL-C levels through the joint effects of apoE2 adhesion and VLDL hydrolysis. Moreover, apoE2 is correlated with low bone mass and bone mineral density (BMD), as reflected by biomarkers of high bone resorption, including a reduced serum ratio of osteoprotegerin/receptor activator of nuclear factor kappa B (NFkB) ligand (OPG/RANKL) in men and high serum C-terminus collagen peptide and urinary deoxypyridinoline levels in postmenopausal women (69, 70).

Vitamin D is a fat-soluble vitamin; the two most important members are exogenous vitamin D2 derived from ergosterol in plants and fungi, and endogenous vitamin D3 derived from 7-dehydrocholesterol (7-DHC) in the skin (71). 7-DHC stored in the human epidermis is irradiated with ultraviolet B (UVB) rays (290–315 nm) in sunlight to produce the vitamin D3 precursor. Both vitamins D2 and pre-D3 are synthesized as blood biomarkers of 25 hydroxyvitamin D (25OHD) in the liver, mainly through equal catalysis by 25 hydroxylase (CYP2R1) (71). Subsequently, 25OHD is converted into the active form of 1,25-dihydroxyvitamin D (1,25 (OH)_2_D) by 25OHD-1α hydroxylase (CYP27B1) or into the inactive form of 24,25-dihydroxyvitamin D (24,25(OH)2D) by 24-hydroxylase (CYP24) in the kidney (71, 72). Circulating 1,25 (OH)_2_D is transported by vitamin D-binding protein (DBP) to target organ tissues such as the intestine, kidneys, and bones (71). After binding with the vitamin D receptor (VDR) in these tissues, 1,25 (OH)_2_D regulates the transcription of target genes, thereby playing a classic role in calcium balance (71). An animal model fed a high-fat and high-cholesterol diet showed that the reduced production of vitamin D could be attributed to the alleviated CYP2R1 expression in the liver induced by increasing circulating cholesterol, glucose, and insulin levels (72). Thus, vitamin D deficiency may occur due to elevated LDL-C levels and bone resorption in patients with FH. The effect of rs7412 (p.Arg176Cys) on vitamin D deficiency require further examination.

### Advantages and limitations

This study has several advantages. First, this is the first human-based study to analyze the cause-and-effect relationship between FH and the risk of multiple vitamin deficiencies. Second, FH showed significant harmful effects on thiamine and vitamin D deficiencies. Third, the results of the leave-one-out sensitivity analysis confirmed the causal relationships between FH and the risk of thiamine and vitamin D deficiencies. Moreover, the SNPs contributing to thiamine and vitamin D deficiencies in FH were identified. However, this study has several limitations. First, Although Bonferroni correction was used to control the occurrence of false positives in the analysis, type I error may be increased by the sample overlapping of exposures and outcomes, both of which were from Finngen consortium in this study, leading to bias in classic MR methods, thus these findings should be interpreted with caution. Second, the causal association between rs112898275 and thiamine deficiency has not been reported yet. The effect of rs112898275 in LDLR region on thiamine deficiency was hypothesized. The actual contribution of rs112898275 in LDLR region for the association between FH and thiamine deficiency will be studied in the future. Third, MR analysis can only calculate the specific OR value without determining the hazard ratio (HR). Future longitudinal studies should use log-rank tests and Cox regression models for HR. Fourth, FH showed no deficiencies in other neurotropic B, vitamin B6, and vitamin B12 (59), The mechanism, which differs from that of vitamin thiamine deficiency in FH, requires clarification in the future. Fifth, another mutation in FH, *ApoB*, did not exert an important effect on thiamine and vitamin D deficiencies. Future studies are needed to elucidate this mechanism, which is distinct from those of *LDLR* and *PCSK9*. Sixth, only causal associations between FH with IHD and vitamin D deficiency were detected, and the prominent locus was not identified by leave-one-out sensitivity analysis. More comprehensive measures are needed to elucidate profound mechanisms that differ from that of FH. Finally, the FinnGen GWAS database has not yet released data on other vitamins such as vitamins C, E, and K, and folic acid, which will be analyzed in the future.

## Conclusion

FH was positively associated with increased risks of thiamine and vitamin D deficiencies, thus revealing a prospective and unfortunate complication of FH.

## Data Availability

The original contributions presented in the study are included in the article/**Supplementary Material**. Further inquiries can be directed to the corresponding author.
